# Effects of the timing of maternal antiretroviral therapy initiation, CD4 count, and HIV viral load on birth outcomes in the Eastern Cape province of South Africa: A secondary data analysis

**DOI:** 10.1371/journal.pone.0308374

**Published:** 2024-09-06

**Authors:** Sisanda Siqithi, Oyewole Christopher Durojaiye, Oladele Vincent Adeniyi

**Affiliations:** 1 Department of Paediatrics, Cecilia Makiwane Hospital, Walter Sisulu University, East London, South Africa; 2 Department of Infection and Tropical Medicine, Sheffield Teaching Hospitals NHS Foundation Trust, Sheffield, South Yorkshire, United Kingdom; 3 Department of Microbiology, University Hospitals of Derby and Burton NHS Foundation Trust, Derby, Derbyshire, United Kingdom; 4 Department of Family Medicine, Cecilia Makiwane Hospital, Walter Sisulu University, East London, South Africa; University of Cape Town, SOUTH AFRICA

## Abstract

**Background:**

Antiretroviral therapy (ART) use during pregnancy is essential to prevent vertical transmission of HIV, but it may also increase the risk of adverse birth outcomes. This study investigated the impact of both maternal HIV infection and the timing of ART initiation on birth outcomes in women living with HIV in South Africa.

**Methods:**

This secondary data analysis examined the dataset from an earlier cohort study involving 1709 pregnant women living with HIV who delivered their babies at three major maternity centres in the Eastern Cape province of South Africa between September 2015 and May 2018. The associations between adverse birth outcomes (stillbirth, preterm birth, very preterm birth, and low birth weight) and the timing of maternal ART initiation, peripartum CD4 count, and HIV viral load were examined using logistic regression analysis.

**Results:**

The observed rates of stillbirth, preterm birth, very preterm birth, and low birth weight were 1.4%, 33.5%, 5.4% and 18.0%, respectively. In the multivariable analysis, low birth weight was associated with ART initiated during the second trimester (adjusted odds ratio [aOR] 1.38; 95% confidence interval [CI], 1.03–1.85), low-level viraemia (21–999 copies/ml) (aOR, 1.62; 95% CI, 1.17–2.22), and high-level viraemia (≥1000 copies/ml) (aOR, 1.66; 95% CI, 1.66–2.38) during the peripartum period. Preterm birth was associated with low-level viraemia (aOR, 1.44; 95% CI, 1.16–1.79) and a CD4 count of less than 200 cells/mm^3^ (aOR, 1.35; 95% CI, 1.01–1.82). Very preterm birth was associated with detectable maternal viraemia.

**Conclusion:**

Adverse birth outcomes are common among pregnant women living with HIV, especially those with unsuppressed viraemia. Clinicians and programme managers should prioritise timeous ART initiation and virological suppression in all pregnant women living with HIV.

## Introduction

The advent of antiretroviral therapy (ART) has significantly reduced the global rate of vertical transmission of the human immunodeficiency virus (HIV). Despite the expansion and success of programmes for the prevention of vertical transmission of HIV in South Africa, introduced nationally in 2002 [1], HIV remains a significant contributor to under-five deaths and morbidity [2], with a reported vertical transmission rate of 3% in 2022 [3].

In 2015, the South African government adopted the World Health Organisation (WHO) Option B+ strategy, which entailed immediate initiation of lifelong ART for all pregnant and breastfeeding women living with HIV, regardless of the disease stage or CD4 cell count [4]. This approach aims to improve the health outcomes of women and their babies by preventing vertical transmission through the reduction of maternal viral load. The treatment for pregnant women living with HIV aligns with the guidelines for ART for people living with HIV and constitutes a woman-centred and rights-based approach to ART delivery [5]. The 2015 South African National Guidelines for the prevention of vertical transmission of HIV recommended a combination of tenofovir (TDF) plus lamivudine (3TC)/emtricitabine (FTC) plus efavirenz (EFV) as the preferred first-line option for adults and adolescents [4]. The guidelines were updated in 2019 [6] and 2023 [7], with the combination of TDF, 3TC and dolutegravir (DTG) now the preferred first-line option, aligning with WHO treatment guidelines [5]. DTG offers rapid viral suppression, a high genetic barrier to resistance and minimal side effects. It also has a low drug interaction profile.

The effects of ART on birth outcomes have been studied over the years. While ART undeniably offers significant benefits in reducing vertical transmission of HIV, like any medication, it carries a potential risk for adverse effects. Most studies have outlined adverse effects such as foetal growth restriction, neonatal mortality, prematurity, low birth weight, small for gestational age, birth defects, viral resistance, mitochondrial toxicity, and haematologic and hepatic abnormalities [8–12].

As South Africa has a substantial population of women of childbearing age living with HIV [3], coupled with a significant burden of neonatal and child morbidity and mortality [2,13], we aimed to evaluate the impact of the timing of initiation of maternal ART, CD4 cell count, and viral load on birth outcomes in the Eastern Cape, South Africa. The findings from our study could offer valuable insights for shaping health policies and planning interventions to prevent the vertical transmission of HIV in South Africa and other similar settings.

## Materials and methods

### Study design and setting

In September 2022, we conducted a secondary analysis of data from the East London Prospective Cohort study [14]. The study was a cross-sectional survey carried out between September 2015 and May 2016 in three major maternity centres in the Buffalo City Metropolitan/Amathole districts of the Eastern Cape province of South Africa. These health facilities collectively serve a population of approximately 1.7 million residents. The national guidelines on the prevention of vertical transmission of HIV were already uniformly implemented across the region at the time of the survey. All pregnant women diagnosed with HIV during their first antenatal visit received adherence counselling and commenced ART in accordance with the national guidelines. The database of the East London Prospective Cohort study contains sociodemographic, clinical and laboratory data, as well as birth outcomes for 1709 women living with HIV who delivered their babies at the maternity centres of the participating hospitals. During the survey period, the prevalence of HIV in the general population and among pregnant women in the region was 12.7% and 30%, respectively [14].

The methodology of the East London Prospective Cohort study has been previously described in detail [14]. The study examined the effectiveness of implementing the WHO Option B+ strategy in preventing the vertical transmission of HIV in the region. The sample size was determined based on the estimated proportion of pregnant women with HIV retained in care one year after delivery in the study population. This resulted in an estimated sample size of 1,709 participants, adjusted for a probable 10% loss to follow-up within the first six months post-delivery, and allowing for a 2.5% margin of error with a 95% confidence level. All pregnant women living with HIV who attended the maternity centres of the selected hospitals during the study period were eligible to participate. Participants were recruited consecutively in the postnatal wards within 24 hours of vaginal delivery or 72 hours of caesarean section delivery. None of the approached participants refused to participate. Trained research assistants conducted face-to-face interviews using a structured questionnaire. Research nurses checked the participants’ viral loads and CD4 counts and drew venous blood from those who did not have a documented viral load and CD4 count within the month of delivery. All viral load assays were performed by the National Health Laboratory Services according to standard protocols. Clinical data were gathered through the interviews, which included information on awareness of HIV serostatus at the first antenatal visit, gestational age at the first antenatal visit (categorised by trimester based on last normal menstrual period and/or obstetric ultrasound), and duration on ART. Adherence to ART was evaluated using self-reports, timely pharmacy refills, and records of ART defaults, verified through medical records.

### Data collection

The following data were extracted from the database: maternal demographics, HIV serostatus at first antenatal visit, maternal ART regimens and duration on ART, CD4 cell count and HIV viral load at delivery, and newborn characteristic (weight, gestation age, and death). The timing of ART initiation was described in gestational weeks; participants who began ART before their last normal menstrual period were considered to have received preconception therapy. The ART regimens at time of delivery were categorised (according to pre-2023 national guidelines) [4] as first-line regimen (TDF/FTC/EFV), other first-line regimen (i.e., two nucleoside reverse transcriptase inhibitors plus one non-nucleoside reverse transcriptase inhibitor [2 NRTIs + NNRTI]), or second line (ritonavir-boosted lopinavir [LPV/r] regimens). Maternal viraemia was categorised as undetectable (HIV viral load ≤ 20 copies/ml), low-level viraemia (21–999 copies/ml), or high-level viraemia (≥ 1000 copies/ml).

### Outcomes and definitions

To assess the effects of maternal HIV characteristics on newborns, the following outcome measures were used: stillbirth (defined as death occurring after 28 weeks of pregnancy but before or during birth, based on the definition of stillbirth in South Africa and the United Nations report on global stillbirth rates) [13]; preterm birth (defined as delivery before 37 completed weeks of gestation); very preterm birth (defined as delivery between 28 and less than 32 weeks of gestation); and low birth weight (defined as birth weight of less than 2500 grams irrespective of gestational age).

### Statistical analysis

Categorical data were presented as frequency counts and percentages, while numerical data were summarised as means with standard deviations (SD) or medians with interquartile ranges (IQR), depending on the degree of skewness in the distributions. Categorical outcome frequencies were compared using the Chi-square test. Logistic regression was used to assess bivariate associations between birth outcomes (stillbirth, very preterm, preterm, and low birth weight) and the timing of maternal ART initiation, peripartum CD4 count, and HIV viral load. Two-sided P-values less than 0.05 were considered statistically significant for all analyses. Only variables with P-values less than or equal to 0.05 were included in the multivariate analysis. The model was adjusted for age, employment status, educational level, alcohol use, smoking, and duration on ART. All covariates were determined a priori. Analyses were performed using Stata version 17 (Stata Corporation, College Station, Texas, USA). In an earlier sensitivity analysis of the East London Prospective Cohort, no statistically significant differences were found in the sociodemographic and clinical characteristics between participants with and without missing birth outcome data [15].

### Ethical considerations

The current study was approved by the Walter Sisulu University (South Africa) Ethics Committee (Reference number: 025/2022) and conducted in accordance with the Helsinki Declaration and local institutional policies on human research. Each participant in the East London Prospective Cohort study gave written informed consent for voluntary participation. Participants under the age of 18 were assisted by their legal guardians and provided assent for their involvement in the study [14]. In our current study, all data from the East London Prospective Cohort study were fully anonymised before being accessed.

## Results

### Maternal characteristics

The study cohort comprised 1709 pregnant women living with HIV, aged between 14 and 47 years, with a mean age of 29.6 (SD, 6.2) years. The majority of the pregnant women were single (69.5%), unemployed (74.7%), resided in semi-urban settings (46.3%), and had two or more children (69.5%). Tables 1 and 2 present the sociodemographic and clinical characteristics of the cohort, respectively. Most of the women (80.9%) living with HIV were aware of their status at the time of their first antenatal visit. In total, 5.5% reported a negative serostatus, and 13.6% had never undergone an HIV test prior to their first antenatal visit. Both of these groups tested positive at first antenatal visit and were initiated on ART on the same day. Among those living with HIV who were aware of their status (*n* = 1392), 72.2% were on already on ART at the time of their first antenatal visit (*n* = 998). The most common ART regimen (84.1%) at the time of delivery was the combination TDF/FTC/EFV (first-line regimen). Among women with available peripartum viral load results (*n* = 1463), undetectable, low-level, and high-level HIV viraemia were recorded in 56.9% (832/1463), 25.2% (369/1463) and 17.9% (262/1463) of participants, respectively. The majority (88.1%; 1298/1474) had a peripartum CD4 count of over 200 cells/mm^3^ (Table 2).

**Table 1:** Sociodemographic of the participants (N = 1709).

Characteristics	n (%)
Age (years), mean ± SD (range)	30 ± 6.2 (14–47)
Marital status	
Married	312 (18.3)
Single	1187 (69.5)
Co-habiting	186 (10.9)
Divorced/separated	24 (1.4)
Place of residence	
Rural	585 (34.2)
Semi-urban	792 (46.3)
Urban	332 (19.4)
Level of education	
No formal education	5 (0.3)
Grade 1–6	115 (6.7)
Grade 7–12	1479 (86.5)
Tertiary	110 (6.4)
Smoking status	
Non-smoker	1529 (89.5)
Smoked during pregnancy	100 (5.9)
Quit during pregnancy	80 (4.7)
Alcohol use	
Never drank	1043 (61.0)
Drank during pregnancy	235 (13.8)
Stopped during pregnancy	431 (25.2)
Employment status	
Unemployed	1277 (74.7)
Employed	432 (25.3)
Parity	
1	521 (30.5)
≥2	1188 (69.5)

Data are presented as n (%) unless otherwise indicated.

SD–standard deviation.

**Table 2:** Maternal clinical characteristics and birth outcomes (N = 1709).

Characteristics	n (%)
***Maternal clinical characteristics***	
Gestational age at first antenatal visit	
First trimester	210 (12.3)
Second trimester	1229 (71.9)
Third trimester	270 (15.8)
Self-reported HIV status at first antenatal visit	
Positive	1382 (80.9)
Negative	94 (5.5)
No prior HIV test	233 (13.6)
Timing of ART initiation	
Preconception	998 (58.4)
First trimester	103 (6.0)
Second trimester	487 (28.5)
Third trimester	121 (7.1)
ART regimen during delivery	
First-line regimen (TDF/FTC/EFV)	1437 (84.1)
Other first-line regimen (2NRTIs + 1 NNRTI)	71 (4.2)
Second-line regimen	35 (2.0)
Not recorded	166 (9.7)
Peripartum viral load, copies/ml	
Undetectable (≤ 20)	832 (48.7)
Low-level viraemia (21–999)	369 (21.6)
High-level viraemia (≥ 1000)	262 (15.3)
Not recorded	246 (14.4)
Peripartum CD4 count, cells/mm^3^	
< 200	176 (10.3)
200–499	739 (43.2)
≥ 500	559 (32.7)
Not recorded	235 (13.8)
***Newborn characteristics***	
Birth outcomes	
Stillbirth	24 (1.4)
Live birth	1676 (98.1)
Not recorded	9 (0.5)
Gestational age (weeks), mean ± SD (range)^a^	37.6 ± 2.7 (26–42)
Full-term birth^a^	1076 (66.5)
Preterm birth^a^	542 (33.5)
Birth weight (grams), median (IQR)^b^	3034 (2649–3400)
Normal birth weight (≥ 2500 g)	1374 (82.0)
Low birth weight (< 2500 g)	302 (18.0)
Birth weight (grams), median (IQR) by gestational age	
Full-term	3200 (2920–3500)
Preterm	2690 (2060–2900)

Data are presented as n (%) unless otherwise indicated.

ART–antiretroviral therapy; EFV–efavirenz; FTC–emtricitabine; HIV–human immunodeficiency virus; IQR, interquartile range; NNRTI–non-nucleoside reverse transcriptase inhibitor; NRTI–nucleoside reverse transcriptase inhibitor; SD—standard deviation; TDF–tenofovir disoproxil fumarate.

^a^ Gestational age was documented in 1618 out of the 1676 live births.

^b^ Birth weight of 1676 live births.

### 1. Newborn characteristics

There were 24 (1.4%; 24/1700) stillbirths. The median birth weight of the recorded 1676 live births was 3034 (IQR, 2649–3400; range, 735–4950) grams. In total, 18.0% (302/1676) had low birth weight. Gestational age was available in 1618 live births, with a mean of 37.6 (SD, 644; range 26–42) weeks. The majority (66.5%; 1076/1618) of babies were delivered at term with a median birth weight of 3200 (IQR, 2920–3500) grams. Of the 542 (33.5%; 542/1618) preterm births, 87 (5.4%; 87/1618) were very preterm (Table 2).

### Association between maternal HIV-related factors and adverse birth outcomes

Logistic regression analysis revealed no significant association between the timing of maternal ART initiation and preterm birth. While ART initiation during the first trimester initially appeared to be positively associated with very preterm birth, this association disappeared after adjusting for age, employment status, educational level, alcohol use, smoking, and duration on ART (Table 3).

**Table 3:** Multivariate analysis of maternal risk factors associated with adverse birth outcomes.

	Stillbirth		Preterm Birth		Very Preterm Birth		Low Birth Weight	
Variable	OR (95% CI)	aOR (95% CI)	OR (95% CI)	aOR (95% CI)	OR (95% CI)	aOR (95% CI)	OR (95% CI)	aOR (95% CI)
Timing of ART initiation								
Preconception	1	1	1	1	1		1	1
First trimester	2.17 (0.61–7.70)	1.91 (0.48–7.68)	0.94 (0.77–1.15)	0.99 (0.80–1.23)	2.80 (1.31–5.99)*	1.56 (0.68–3.60)	1.83 (1.11–2.30)*	1.53 (0.92–2.56)
Second trimester	0.87 (0.33–2.28)	0.76 (0.28–2.08)	0.95 (0.87–1.04)	0.97 (0.88–1.06)	1.56 (1.00–2.43)	1.52 (0.95–2.44)	1.49 (1.13–1.96)*	1.38 (1.03–1.85)*
Third trimester	0.57 (0.07–4.40)	1.14 (0.13–9.89)	0.97 (0.84–1.13)	0.98 (0.83–1.14)	0.94 (0.39–2.25)	1.17 (0.47–2.95)	1.32 (0.82–2.13)	1.11 (0.68–1.81)
Peripartum maternal viral load, copies/ml								
Undetectable (≤ 20)	1	1	1	1	1	1	1	1
Low-level viraemia (21–999)	0.14 (0.02–1.04)	0.16 (0.02–1.6)	1.46 (1.17–1.80)**	1.44 (1.16–1.79)**	2.26 (1.35–3.79)**	2.29 (1.37–3.85)**	1.57 (1.15–2.14)*	1.62 (1.17–2.22)*
High-level viraemia (≥ 1000)	0.78 (0.26–2.36)	1.00 (0.34–2.92)	1.30 (1.01–1.68)*	1.25 (0.96–1.63)	3.24 (1.89–5.54)***	3.34 (1.93–5.78)***	1.76 (1.25–2.48)*	1.66 (1.16–2.38)*
Peripartum maternal CD4 count, cells/mm^3^								
≥ 500	1	1	1	1	1	1	1	1
200–499	1.19 (0.46–3.10)	1.23 (0.47–3.22)	1.08 (0.88–1.32)	1.09 (0.89–1.33)	0.70 (0.44–1.11)	0.71 (0.44–1.14)	1.00 (0.75–1.34)	1.04 (0.78–1.40)
< 200	0.90 (0.19–4.37)	1.19 (0.23–6.08)	1.30 (0.97–1.74)	1.35 (1.01–1.82)*	1.10 (0.57–2.21)	1.19 (0.61–2.33)	1.25 (0.82–1.91)	1.37 (0.89–2.12)

ART–antiretroviral therapy; aOR–adjusted odds ratio; CI–confidence interval.

*P < 0.05.

** *P* < 0.01.

*** *P* < 0.001.

Preterm birth was associated with low-level peripartum maternal viraemia (adjusted odds ratio [aOR], 1.44; 95% confidence interval [CI], 1.16–1.79; *p* < 0.01) and a peripartum maternal CD4 count of less than 200 cells/mm^3^ (aOR, 1.35; 95% CI, 1.01–1.82). However, there was no association between peripartum CD4 count and very preterm birth. Both low-level (aOR, 2.29; 95% CI, 1.37–3.85) and high-level (aOR, 3.34; 95% CI, 1.93–5.78) maternal viraemia were associated with a higher risk of very preterm birth. No significant differences were detected in the association between peripartum maternal viraemia and birth outcomes, regardless of whether adjustments were made for maternal CD4 count.

There were no significant associations between stillbirth and the timing of maternal ART initiation, peripartum HIV viral load, and CD4 count. The initiation of ART during the second trimester was associated with a higher risk of low birth weight (aOR, 1.38; 95% CI, 1.03–1.85) than initiation of ART pre-conception. Similarly, low-level (aOR, 1.62; 95% CI, 1.17–2.22) and high-level (aOR, 1.66; 95% CI, 1.66–2.38) maternal viraemia were associated with low birth weight (Table 3).

## Discussion

This current study sought to examine the birth outcomes of HIV-exposed neonates delivered to women receiving ART in the Eastern Cape, South Africa. In addition, it investigated the effects of peripartum maternal CD4 cell count, viral load, and the timing of maternal ART initiation on adverse birth outcomes. We observed several adverse birth outcomes within the cohort: stillbirths occurred in 1.4% of cases, very preterm births in 5.4%, preterm births in 33.5%, and low birth weight in 18% of all newborns.

The rate of stillborn infants in the cohort is within the South Africa’s national average of 13.9–18.9 stillbirths per 1000 total births and similar to the global average of 13.9 stillbirths per 1000 total births [13]. Consistent with other studies [16,17], the risk of stillbirth in our study did not differ significantly between women who took ART preconception and those who initiated ART after conception. While a low maternal CD4 count has been linked to an increased risk of stillbirth due to poor maternal immune status [17–19], we did not observe this association in our study. This lack of association in our cohort may be partly attributed to the low number of stillbirth events.

Studies on low birth weight and prematurity in HIV-exposed neonates have occasionally shown conflicting findings as a result of numerous influencing factors such as maternal socioeconomic status, age, ethnicity, preeclampsia, cervical incompetence, and smoking [9]. However, maternal HIV viral load and CD4 count, as well as exposure to ART, have consistently been linked with low birth weight and preterm birth [8,9,20–23]. We found higher rates of low birth weight and prematurity compared to South Africa’s national averages of 16.6% for low birth weight and 13% for preterm birth [24]. However, these findings are consistent with other similar studies on HIV-exposed newborns [9,20–23,25].

The effects of the timing of maternal ART exposure on low birth weight is not clear. While some studies have reported an increased risk of low birth weight in children exposed to ART since conception [21,25], others have not found any association [9,16]. However, in our study, we observed that ART initiated during the second trimester was associated with low birth weight. The reason for this observation in our cohort is not clear. We also found a link between detectable maternal HIV viral load and low birth weight. Uncontrolled maternal viral replication can lead to low birth weight through various and complex mechanisms, including dysregulation of the maternal immune response, direct viral effects on foetal development, maternal health complications, and opportunistic infections [26,27].

Furthermore, we found that pregnant women who had detectable HIV viral loads and a CD4 count lower than 200 cells/mm3 were more likely to have delivered premature babies. Low peripartum CD4 count has also been linked to preterm birth in similar studies [8,20]. In our study, women with an HIV viral load of ≥ 1000 copies/ml were three times more likely to give birth to very preterm babies than those with undetectable viral loads. Evidence has explicitly shown that viral suppression with ART is an effective strategy to significantly reduced HIV transmission at the population level [16,28,29].

Although all pregnant women in our cohort were taking ART in accordance with national guidelines [4], the 48.7% rate of undetectable viral load is suboptimal for achieving the goals of programmes aimed at preventing vertical transmission of HIV. Achieving undetectable viraemia should be the primary goal for all pregnant women living with HIV. Non-suppressed HIV viral load in pregnant women should be considered an obstetric emergency, in order to prevent vertical transmission of HIV. It should be noted that in our cohort, all parturient women with high-level viraemia and their babies were managed in accordance with the national guidelines for preventing vertical transmission of HIV [4]. Undoubtedly, a comprehensive approach is required to address potential risk factors for persistent vertical transmission in the country, such as late HIV diagnosis and delayed initiation of ART among pregnant mothers [30].

Some limitations of this study should be acknowledged. The study was conducted in few selected facilities within one province in South Africa and was limited to a specific timeframe. As such, the findings may not be fully representative of the entire country. However, the three health facilities chosen for the study reflect the diverse demographics and levels of healthcare in the province and country. Furthermore, we did not consider the influence of unrecorded confounders or examine the effects of non-HIV risk factors for adverse birth outcomes. For instance, data on other congenital infections, obstetric complications, anaemia, and maternal malnutrition, which are common in sub-Saharan Africa [27], were not captured in the study database. We also did not assess the impact of different ART regimens owing to the low number of patients on non-first-line therapies. Although, gestational age was determined using self-reported menstrual history and/or obstetric ultrasound, there is a possibility that the accurate gestational age was not recorded, especially for participants whose first antenatal visit occurred late in pregnancy. However, categorising gestational age by trimester could have minimised this inaccuracy. Lastly, a comparison of birth outcomes with mothers without HIV would have enriched the findings of the study. Future studies should aim to enrol all newborns in the region for comparison.

## Conclusions

Adverse birth outcomes are common in pregnant women infected with HIV, especially those with unsuppressed viraemia. Clinicians and programme managers should prioritise timeous ART initiation and virological suppression in all pregnant women living with HIV. It is advisable for experienced clinicians to oversee the delivery of pregnant women living with HIV who have low peripartum CD4 counts and high HIV viral loads to optimise birth outcomes.

## References

[1] National Department of Health. Policy and guidelines for the implementation of the PMTCT programme. Pretoria: NDOH; 2008. Available from: https://www.gov.za/sites/default/files/gcis_document/201409/pmtct-policy-and-guidelines.pdf (accessed 28 January 2024).

[2] Bamford LJ, McKerrow NH, Barron P, Aung Y. Child mortality in South Africa: fewer deaths, but better data are needed. *S Afr Medical J*. 2018; 108 (3 Suppl 1): S25–S32. doi: 10.7196/SAMJ.2017.v108i3b.12779

[3] Joint United Nations Programme on HIV/AIDS. UNAIDS data 2023. Geneva: UNAIDS; 2023. Available from: https://www.unaids.org/sites/default/files/media_asset/data-book-2023_en.pdf (accessed 3 February 2024).

[4] National Department of Health. National consolidated guideline for the prevention of mother-to-child transmission of HIV (PMTCT) and the management of HIV in children, adolescent and adults. Pretoria: NDOH; 2015. Available from: https://sahivsoc.org/Files/ART%20Guidelines%2015052015.pdf (accessed 18 January 2024).

[5] World Health Organisation (WHO). Consolidated guidelines on HIV prevention, testing, treatment, service delivery and monitoring: recommendations for a public health approach. Geneva: WHO; 2021. 34370423

[6] National Department of Health. 2019 ART clinical guidelines for the management of HIV in adults, pregnancy, adolescents, children, infants and neonates. Pretoria: NDOH; 2019. Available from: https://www.health.gov.za/wp-content/uploads/2020/11/2019-art-guideline.pdf (accessed 20 January 2024).

[7] National Department of Health. 2023 ART clinical guidelines for the management of HIV in adults, pregnancy and breastfeeding, adolescents, children, infants and neonates. Pretoria: NDOH; 2023. Available from: www.differentiatedservicedelivery.org/wp-content/uploads/National-ART-Clinical-Guideline-2023_04_28-signed.pdf (accessed 20 January 2024).

[8] Uthman OA, Nachega JB, Anderson J, Kanters S, Mills EJ, Renaud F, et al. Timing of initiation of antiretroviral therapy and adverse pregnancy outcomes: a systematic review and meta-analysis. *Lancet HIV*. 2017; 4(1): e21–e30. doi: 10.1016/S2352-3018(16)30195-3 27864000

[9] Delicio AM, Lajos GJ, Amaral E, Cavichiolli F, Polydoro M, Milanez H. Adverse effects in children exposed to maternal HIV and antiretroviral therapy during pregnancy in Brazil: a cohort study. *Reprod Health*. 2018; 15(1):76. doi: 10.1186/s12978-018-0513-8 29747664 PMC5946413

[10] Ciaranello AL, Seage GR 3rd, Freedberg KA, Weinstein MC, Lockman S, Walensky RP. Antiretroviral drugs for preventing mother-to-child transmission of HIV in sub-Saharan Africa: balancing efficacy and infant toxicity. AIDS. 2008; 22(17): 2359–69. doi: 10.1097/QAD.0b013e3283189bd7 18981776 PMC2881583

[11] Senise JF, Castelo A, Martínez M. Current treatment strategies, complications and considerations for the use of HIV antiretroviral therapy during pregnancy. AIDS Rev. 2011; 13(4):198–213. 21975356

[12] Newell ML, Bunders MJ. Safety of antiretroviral drugs in pregnancy and breastfeeding for mother and child. Curr Opin HIV AIDS. 2013; 8(5): 504–10. doi: 10.1097/COH.0b013e3283632b88 . 23743789

[13] UN IGME. Never forgotten–the situation of stillbirth around the globe. Report of the United Nations Inter-agency Group for Child Mortality Estimation (UN IGME), 2022. UNICEF: New York; 2023.

[14] Adeniyi OV, Ajayi AI, Selanto-Chairman N, Goon DT, Boon G, Fuentes YO, et al. East London Prospective Cohort Study (ELPCS) Group. Demographic, clinical and behavioural determinants of HIV serostatus non-disclosure to sex partners among HIV-infected pregnant women in the Eastern Cape, South Africa. PLoS One. 2017; 12(8):e0181730. doi: 10.1371/journal.pone.0181730 28837563 PMC5570311

[15] Adeniyi OV, Obi CL, Goon DT, Iweriebor B, Selanto-Chairman N, Carty C, et al. Factors associated with peripartum virologic suppression in Eastern Cape Province, South Africa: a retrospective cross-sectional analysis. Clin Infect Dis. 2021; 73(10): 1750–8. doi: 10.1093/cid/ciab206 33677576 PMC8599206

[16] Mandelbrot L, Tubiana R, Le Chenadec J, Dollfus C, Faye A, Pannier E, et al; ANRS-EPF Study Group. No perinatal HIV-1 transmission from women with effective antiretroviral therapy starting before conception. Clin Infect Dis. 2015; 61(11): 1715–25. doi: 10.1093/cid/civ578 26197844

[17] Favarato G, Townsend CL, Peters H, Sconza R, Bailey H, Cortina-Borja M, et al. Stillbirth in women living with HIV delivering in the United Kingdom and Ireland: 2007–2015. J Acquir Immune Defic Syndr. 2019; 82(1):9–16. doi: 10.1097/QAI.0000000000002087 31149953

[18] Chi BH, Wang L, Read JS, Taha TE, Sinkala M, Brown ER, et al. Predictors of stillbirth in sub-Saharan Africa. Obstet Gynecol. 2007; 110(5): 989–97. doi: 10.1097/01.AOG.0000281667.35113.a5 17978109

[19] Aminu M, Unkels R, Mdegela M, Utz B, Adaji S, Van den Broek N. Causes of and factors associated with stillbirth in low- and middle-income countries: a systematic literature review. BJOG. 2014; 121 (Suppl 4):141–53. doi: 10.1111/1471-0528.12995 25236649

[20] Chen JY, Ribaudo HJ, Souda S, Parekh N, Ogwu A, Lockman S, et al. Highly active antiretroviral therapy and adverse birth outcomes among HIV-infected women in Botswana. J Infect Dis. 2012; 206(11): 1695–705. doi: 10.1093/infdis/jis553 23066160 PMC3488194

[21] Li N, Sando MM, Spiegelman D, Hertzmark E, Liu E, Sando D, et al. Antiretroviral therapy in relation to birth outcomes among HIV-infected women: a cohort study. J Infect Dis. 2016; 213(7): 1057–64. doi: 10.1093/infdis/jiv389 26265780

[22] Elenga N, Djossou FÉL, Nacher M. Association between maternal human immunodeficiency virus infection and preterm birth: a matched case-control study from a pregnancy outcome registry. Medicine (Baltimore). 2021; 100(4): e22670. doi: 10.1097/MD.0000000000022670 33530154 PMC7850744

[23] Fentie EA, Yeshita HY, Bokie MM. Low birth weight and associated factors among HIV positive and negative mothers delivered in northwest Amhara region referral hospitals, Ethiopia, 2020 a comparative cross-sectional study. PLoS One. 2022; 17(2): e0263812. doi: 10.1371/journal.pone.0263812 35148350 PMC8836330

[24] Ohuma EO, Moller AB, Bradley E, Chakwera S, Hussain-Alkhateeb L, Lewin A, et al. National, regional, and global estimates of preterm birth in 2020, with trends from 2010: a systematic analysis. Lancet. 2023; 402(10409):1261–1271. doi: 10.1016/S0140-6736(23)00878-4 37805217

[25] Kreitchmann R, Li SX, Melo VH, Fernandes Coelho D, Watts DH, Joao E, et al. Predictors of adverse pregnancy outcomes in women infected with HIV in Latin America and the Caribbean: a cohort study. BJOG. 2014; 121(12):1501–8. doi: 10.1111/1471-0528.12680 24602102 PMC4157114

[26] Faye A, Pornprasert S, Mary JY, Dolcini G, Derrien M, Barre-Sinoussi F, et al; ANRS 1267 study team and the HIV-1 PMTCT-PlaNet. Characterization of the main placental cytokine profiles from HIV-1-infected pregnant women treated with anti-retroviral drugs in France. Clin Exp Immunol. 2007; 149(3):430–9. doi: 10.1111/j.1365-2249.2007.03411.x 17511776 PMC2219329

[27] Zenebe A, Eshetu B, Gebremedhin S. Association between maternal HIV infection and birthweight in a tertiary hospital in southern Ethiopia: retrospective cohort study. Ital J Pediatr. 2020; 46(1):70. doi: 10.1186/s13052-020-00834-3 32448252 PMC7247191

[28] Fowler MG, Qin M, Fiscus SA, Currier JS, Flynn PM, Chipato T, et al; IMPAACT 1077BF/1077FF PROMISE Study Team. Benefits and risks of antiretroviral therapy for perinatal HIV prevention. N Engl J Med. 2016; 375(18): 1726–37. doi: 10.1056/NEJMoa1511691 27806243 PMC5214343

[29] Das M, Chu PL, Santos GM, Scheer S, Vittinghoff E, McFarland W, et al. Decreases in community viral load are accompanied by reductions in new HIV infections in San Francisco. PLoS One. 2010; 5(6): e11068. doi: 10.1371/journal.pone.0011068 20548786 PMC2883572

[30] Naidoo K, Hoque M, Buckus S, Hoque M, Jagernath K. Prevention-of-mother-to-child-transmission (PMTCT) program outcomes in South Africa in the pre-COVID and COVID eras. BMC Public Health. 2023; 23(1):1395. doi: 10.1186/s12889-023-16214-5 37474920 PMC10357590

